# Knowledge structure and theme trends analysis on general practitioner research: A Co-word perspective

**DOI:** 10.1186/s12875-016-0403-5

**Published:** 2016-01-29

**Authors:** Yang Hong, Qiang Yao, Ying Yang, Jun-jian Feng, Shu-de Wu, Wen-xue Ji, Lan Yao, Zhi-yong Liu

**Affiliations:** 1 School of Medicine and Health Management, Tongji Medical College, Huazhong University of Science and Technology, Wuhan, 430030 Hubei China; 2 School of Political Science and Public Administration, Wuhan University, Wuhan, 430072 Hubei China

**Keywords:** General practitioners, Research trends, Co-word analysis, Social network analysis, Knowledge map

## Abstract

**Background:**

General practitioners (GPs) are the most important providers of primary health care, as proven by related research published several decades ago. However, the knowledge structure and theme trends of such research remain unclear. Accordingly, this study aimed to provide an overview of the development of research on GPs over the period of 1999 to 2014.

**Methods:**

Studies on GPs conducted from 1999 to 2014 were retrieved from PubMed. In this work, co-word, social network analysis, and theme trends analyses were conducted to reveal the knowledge structures and thematic evolution of research on GPs.

**Results:**

The number of conducted studies on GPs increased. However, growth speed slowed down during the past 16 years. A total of 27 high-frequency keywords were identified in 1999 to 2003, and more new and specific high-frequency keywords emerged in the subsequent periods. The dynamic of this field was first divergent and then considered convergent. Specifically, network centralization is 19.77 %, 19.09 %, and 13.04 % in 1999 to 2003, 2004 to 2008 and 2009 to 2014, respectively. The major topics of research on GPs completed from 1999 to 2014 were “physician/family,”“attitude of health personnel,” and “primary health care,” and “general practitioner” communities, and so on.

**Conclusion:**

The research themes on GPs are relatively stable at the beginning of the 21^st^ century. However, the thematic evolution and research topics of research on GPs are changing dynamically in recent years. Themes related to the roles and competencies of GPs, and the relations between general practitioner and patients/others have become research foci on GPs. In addition, more substantial research especially on comprehensive approaches and holistic modeling, which have been defined in the European Definition of General Practice/Family Medicine, are expected to be accomplished.

## Background

High-quality primary care is the foundation of effective and efficient health care systems. The essential elements of the practice of primary care include accessibility as the first-contact point of entry to the health care system, continuity, comprehensiveness, coordination of referrals, and understanding of the family and community context of health [1–3]. General practice is a key discipline of primary care, and in many countries, general practitioners (GPs) are physicians who are directly accessible to the public. Thus, strengthening the knowledge structure and analyzing theme trends in GPs will contribute to the provision of better health care for all [4, 5].

Historically, the role of a GP was once performed by any doctor qualified in a medical school working in the community [6]. However, since the 1950s, general practice has become a specialty in its own right, with specific training requirements tailored to each country. The Alma Ata Declaration in 1978 set the intellectual foundation of what primary care and general practice should be [7–9]. Currently, GPs are specialist physicians trained in the principles of the discipline [10]. They are personal doctors who are responsible primarily for the provision of comprehensive and continuing care to every individual seeking medical care irrespective of age, sex, and illness. GPs care for individuals in the context of their family, their community and their culture, always respecting the autonomy of their patients [11–13]. The core competencies of GPs are primary care management, person-centered care, specific problem solving skills comprehensive approach, community orientation, and holistic modeling. However, the role of a GP can vary greatly between (or even within) countries [14]. In urban areas of developed countries, their roles tend to be narrower and focused on the care of chronic health problems, treatment of acute non-life-threatening diseases, early detection and referral to specialized care of patients with serious diseases, and preventative care including health education and immunization. However, in developing countries or in the rural areas of developed countries, a GP may be routinely involved in pre-hospital emergency care, the delivery of babies, community hospital care, and performance of low-complexity surgical procedures. Moreover, in some healthcare systems, GPs work in primary care centers where they play a central role in the healthcare team, whereas GPs can work as single-handed practitioners in other models of care [15, 16].

Entering the 21^st^ century, the connotation and role of GPs was also developed with changes of society and health reform all over the world. In particular, GPs not only provide comprehensive and compassionate health care services in the context of individual needs, their families and communities, but also play a vital role in reducing health inequalities and in delivering high-quality and cost-effective care. The role of GPs as primary care physicians in health risk factor interventions has been well introduced in the literature [17–20]. Many researchers have suggested that GPs can contribute to reducing the prevalence of smoking or alcohol misuse. Moreover, GPs encourage lifestyle changes, especially in nutrition and physical activities. Patients primarily obtain worthy information on nutrition or physical activity from GPs [21]. Hence, to understand the knowledge structure and theme trends on general practitioners further, we use co-word analysis and related technologies in this paper to reveal the research evolution and trends of major themes and knowledge structure on GPs, probe features of the major themes and its development process, and provide an overview of the development trends in the field of GPs during 1999–2014 based on the PubMed database [22–25].

## Methods

### Data source

Data were retrieved and downloaded from PubMed, a biomedical literature database developed by the US National Center for Biotechnology Information. PubMed was selected as the data source for two reasons. First, PubMed is a free authoritative medical literature database consisting of over 25 million citations for biomedical literature from MEDLINE, life science journals, and online books, including the fields of biomedicine and health, covering portions of the life sciences, behavioral sciences, chemical sciences, and bioengineering. Second, the articles from PubMed are indexed with Medical Subject Headings (MeSH) terms, which comprises a set of normalized words that can reflect the contents of articles [26, 27]. The PubMed database and MeSH terms provide a good possibility of extracting emerging keywords. The MeSH terms “family, physician” and “general practitioner” are two different terms in the database. The meanings of the two subject terms are also different and reflect the development of GPs. In this study, retrieval strategies employed included “general practitioners” [MeSH] or “physicians, family” [MeSH terms] according to the entry words, which include “general practitioner,” “practitioner, general,” “practitioners, general,” “physicians, general practice,” “general practice physician,” “general practice physicians,” “physician, general practice,” and “practice physicians, general.” The publication scope was limited to within 1999–2014 and a total of 10704 articles were retrieved on August 7, 2015.

### Scientometrics

Scientometrics is a discipline of measuring science and the effects of scientific work, and its indicators are equally suitable for macro-analysis and micro studies [30]. Scientometrics has been widely used in the fields of data mining, machine learning, and information retrieval [31, 32].

Co-word analysis is a content analysis technique that is based on the assumptions that a scientific field can mark literature and reflect its core contents by abstracting a set of single words, and that the keywords of scientific publications can be treated as signal-words [33]. This technique means that the more frequent the co-occurrence of a pair of words in the literature, the more similar the themes they indicate. Moreover, the frequency of word occurrence in the entire body of a selected field can reflect important themes, and co-occurrence of multiple terms in the same literature that reflects the themes to which they refer [31].

Social network analysis is a method that aims to study the relationship between a set of actors and views social relationships in terms of network theory that consists of nodes (representing words in this study) and ties (represent word relationships in this study) [22, 34, 35]. Centrality is an important index for analyzing the network and determining the influence of a node in the network, including degree centrality, betweenness centrality, and closeness centrality [36]. Degree represents the number of ties to others, while in a friendship network, degree may translate to gregariousness or popularity. Betweenness indicates how frequently a node lies along the geodesic pathways of other nodes in the network and, therefore is an inherently asymmetric measure. Closeness represents the graph-theoretic distance of a given node to all other nodes, and network centralization reflects the whole network tightness.

Thematic evolution analysis [37, 38] was used to detect and visualize the topic evolution of GPs as it can be used to conduct sufficient analyses than co-word networks [39]. The alluvial diagram based from the geographic domain and proposed by Rosvall and Berstrom [40] was also employed in this study to visualize the evolution of networks. The overall evolution provides insights on the evolution of different topics.

Science mapping analysis is an important research topic in the field of scientometrics, and is focused on monitoring a scientific field and delimiting research areas to determine its cognitive structure and its evolution, thereby revealing the hidden key relations among documents, authors, institutions, and topics. The workflow of science mapping analysis contains eight aspects: data retrieval, preprocessing, network extraction, normalization, mapping, analysis, visualization, and interpretation [41–43].

### Data analysis

The process includes mainly three stages: data processing, theme structure, and theme evolution. First, the retrieved articles were downloaded as XML files and imported into the Bibliographic Item Co-Occurrence Matrix Builder (BICOMB) [28] software. Next, the major MeSH terms were extracted and assessed with the BICOMB. The high-frequency MeSH terms were identified and divided into three time periods, and a word occurrence matrix was constructed to support further co-word analysis. The high-frequency words were defined by using the following formula proposed by Donohue in 1973: $N=1/{2}^{\ast}\left(-1+\sqrt{1+8*{I}_1}\right)$ [29]. In this formula, *I*_1_ represents the number of words that occurred once in the articles, and words with higher frequency than *N* were considered high-frequency words. Second, the word occurrence matrix was imported into Ucinet software and visualized based on co-word theory, after which the social network analysis method was used to analyze the themes and knowledge structure of GPs in different time periods. Third, the thematic evolution analysis method and NEViewer software were used to analyze and forecast the theme evolution trends by combining the different stages information. Based on the above analysis, BICOMB [28], Ucinet [44], and NEViewer [39] software were used to analyze the publications for knowledge mapping.

### Growth rate

The entire data were divided into three periods, namely, 1999 to 2003, 2004 to 2008 and 2009 to 2014, to display the research hotspots and developments of research on GPs. The growth rate of the number of publications was determined through the absolute increase of publications and then measured by using two related parameters: relative growth rate (RGR) and double time (Dt) [45, 46].

RGR in the classical growth analysis is defined as

$$ RGR=\left( \ln N2- \ln N1\right)/\left(t2-t1\right), $$

where *N*2 and *N*1 are the cumulative publications in two years, namely, *t*2 and *t*1, respectively. In the present analysis *t*2–*t*1 is taken as one year. Accordingly, RGR can be expressed as RGR = ln (*N*2/*N*1).

*Dt* is the time required for publications to double in number for a given RGR, and is expressed as

$$ Dt=\left(t2-t1\right)* \ln 2/\left( \ln N2- \ln N1\right)= \ln 2/RGR, $$

where *Dt* is a characteristic time for this exponential growth, and a constant value for RGR in each subsequent year indicates that the growth rate is exponential. For example, if the number of articles or pages of a subject doubles in one year then the difference between the logarithms of numbers at the beginning and end of this period must be the logarithm of the number 2. Hence, if the natural logarithm is used, the RGR = 0.693(ln2 ≈ 0.693) and Dt =1.

## Results

The entire set of data was divided into three periods, namely, 1999 to 2003, 2004 to 2008 and 2009 to 2014, to display the hotspots and developments of research on GPs clearly. Table 1 indicates that the high-frequency major MeSH terms, with less than 3 % proportion, have a total frequency accounting for approximately 50 % of the total frequency of major MeSH terms. All these high-frequency MeSH terms provide the hot topics analyzed in substantial research on GPs.

**Table 1:** Descriptive statistics for each measure of research on GP conducted from 1999–2014

Periods	Total papers	Total major MeSH terms	Total frequency of major MeSH terms	Total major MeSH terms with high frequency (*n*, %)	Total frequency of major MeSH terms with high frequency (*n*, %)	*N*
1999 to 2003	2453	1362	9492	27 (1.98)	4583 (48.28)	34.57
2004 to 2008	3521	1792	13989	41 (2.29)	7061 (50.47)	39.43
2009–2014	4729	2093	19214	64 (3.06)	10366 (53.95)	41.83
Total	10703	5247	42695	132 (2.52)	22010 (51.76)	——

Note: *N* is the high-frequency words threshold, words with higher frequency than *N* were considered high-frequency words

**Fig. 1:**
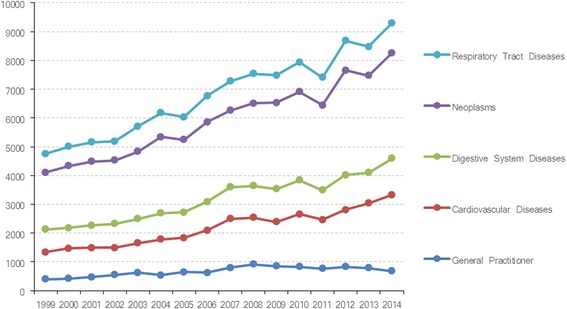
Papers on general practitioners and related disciplines published from 1999–2014 in WoS

Figure 1 reveals the publication trend of research on GPs conducted all over the world from 1999–2014. The figure shows that considerable research on GPs has been well developed since 1999, with 392 records. The number of papers annually increased from 1999–2003 with a period of slow growth. Subsequently, the number of papers continued to increase rapidly and attained a peak in 2008, although a slight decline occurred in 2004. However, a fluctuating decline trend was observed after 2008 (for details, see Table 2).

**Table 2:** Worldwide publication trends of research on GPs

Year	Records	Cumulative	RGR	Dt
1999	392	392	—	—
2000	414	806	0.72	0.96
2001	474	1280	0.46	1.50
2002	550	1830	0.36	1.94
2003	623	2453	0.29	2.37
2004	535	2988	0.20	3.51
2005	644	3632	0.20	3.55
2006	627	4259	0.16	4.35
2007	798	5057	0.17	4.04
2008	918	5975	0.17	4.16
2009	846	6821	0.13	5.23
2010	825	7646	0.11	6.07
2011	765	8411	0.10	7.27
2012	831	9242	0.09	7.36
2013	788	10030	0.08	8.47
2014	674	10704	0.07	10.66

Note: Records: the number of articles published in a specific year; Cumulative: the cumulative number of articles published from 1999 to a specific year; RGR: relative growth rate; Dt: double time

As shown in Table 2, for the last 16 years, the worldwide RGR achieved an average value of 0.22 and an average *D*t of 4.76. Moreover, RGR dropped from 0.72 in 2000 to 0.16 in 2006, but slightly increased to 0.17 in 2007 and 2008. The RGR continuously dropped until it attained a value of 0.07 in 2014. The *D*t reflected a similar trend, in which it increased from 0.96 in 2000 to 10.66 in 2014. These findings suggest that the growth speed of GP-related publications has slowed down in the 16 years. This specific result conforms to the outcomes presented in Fig. 1.

As shown in Fig. 1, although research on GPs has increased during last decades, compared with other fields (e.g., cardiovascular diseases, digestive system diseases, neoplasms and respiratory tract diseases), the gaps between GPs and others has become much larger. In 1999, GP-related papers accounted for 0.08 % of all papers in PubMed and reached a peak in 2008 (0.12 %). Then, the growth speed of GP-related research became increasingly lower compared with other fields and the entire field of medicine. The results conform to the outcomes presented in Table 2.

### Knowledge structure and research topic analysis

The threshold for generating edges was set as five times of co-occurrences, in order to eliminate the weak relation among the major MeSH terms. Keyword centrality was measured by the degree, betweenness, and closeness centrality. Network centralization was applied to analyze the network structure. Specific data are shown in Table 3. As shown in Table 3, the network centralization decreased with the development of GPs, while the mean values of degree and betweenness increased rapidly. The results suggest that the topics in this field are becoming increasingly richer and no longer based on only several words or themes; moreover, he links with topics have become even closer.

**Table 3:** Individual centrality and network centralization of GP research from 1999–2014

Centralization		1999 to 2003	2004 to 2008	2009 to 2014
Degree	Mean ± SD	266.00 ± 417.66	290.05 ± 516.42	300.56 ± 477.66
	Min	66.00	54.00	62.00
	Max	2160.00	3239.00	2561.00
Betweenness	Mean ± SD	3.93 ± 3.54	7.54 ± 7.27	14.19 ± 16.86
	Min	0.37	0.87	0.69
	Max	11.15	25.87	59.90
Closeness	Mean ± SD	33.85 ± 4.55	55.07 ± 7.08	91.38 ± 12.53
	Min	26.00	40.00	63.00
	Max	41.00	65.00	111.00
Network Centralization (%)		19.77	19.09	13.04

Note: *Min* Minimum value, *Max* Maximum value, *SD* Standard Deviation

### Knowledge structure of the time period from 1999–2003

**Fig. 2:**
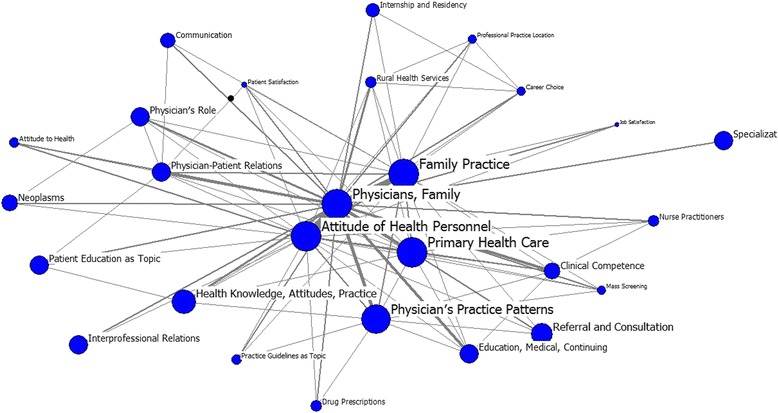
Map for keywords in GP research, 1999–2003.The size of nodes indicates the keywords centrality, and the thickness of the lines indicates the co-occurrence frequency of keywords pairs

A sum of 27 high-frequency keywords were identified from the research on GPs published from 1999–2003 (Table 4). “Physicians, family” with a frequency of 1759 ranks first. This keyword has a degree value of as high as 2160, indicating that it has a direct link with many keywords and is in the central position in the social network of GP-related research. Moreover, keywords “attitude of health personnel,” “family practice,” “physician’s practice patterns,” and “primary health care” contain degree values that rank in the top 5. The betweenness value in Table 4 also shows that these four keywords mentioned above with the same value (11.15), rank top in the list. Meanwhile, these four keywords have the lowest closeness value (26) in the list, indicating that they have the shortest path when they communicate with other keywords. Such a finding indicates that these keywords can transfer information with less dependence on other keywords. Figure 2 demonstrates that the research themes in this period focus mainly on “family practice,” “primary health care,” “attitude of health personnel,” and “physician’s practice patterns.”

**Table 4:** Individual centrality of GP research from 1999–2003

Rank	Major MeSH Terms	Frequency	Percentage (%)	Degree	Betweenness	Closeness
1	Physicians, Family	1759	18.53	2160.00	11.15	26.00
2	Attitude of Health Personnel	463	4.88	871.00	11.15	26.00
3	Family Practice	425	4.48	674.00	11.15	26.00
4	Primary Health Care	275	2.90	415.00	11.15	26.00
5	Physician’s Practice Patterns	233	2.45	444.00	9.77	27.00
6	Physician–Patient Relations	154	1.62	253.00	3.70	33.00
7	Clinical Competence	121	1.27	215.00	2.43	34.00
8	Education, Medical, Continuing	121	1.27	196.00	2.82	33.00
9	Physician’s Role	110	1.16	176.00	4.18	33.00
10	Health Knowledge, Attitudes, Practice	94	0.99	184.00	5.11	30.00
11	Rural Health Services	80	0.84	161.00	3.02	37.00
12	Referral and Consultation	76	0.80	138.00	4.63	31.00
13	Practice Guidelines as Topic	71	0.75	103.00	1.02	38.00
14	Nurse Practitioners	50	0.53	103.00	1.12	37.00
15	Attitude to Health	49	0.52	97.00	1.43	38.00
16	Mass Screening	48	0.51	92.00	1.43	39.00
17	Patient Satisfaction	48	0.51	86.00	0.83	40.00
18	Interprofessional Relations	45	0.47	79.00	3.56	33.00
19	Drug Prescriptions	45	0.47	77.00	1.11	37.00
20	Internship and Residency	44	0.46	88.00	2.22	36.00
21	Communication	42	0.44	94.00	1.52	35.00
22	Specialization	41	0.43	68.00	4.21	33.00
23	Professional Practice Location	39	0.41	103.00	1.29	39.00
24	Job Satisfaction	39	0.41	66.00	0.37	41.00
25	Patient Education as Topic	39	0.41	74.00	2.07	33.00
26	Neoplasms	36	0.38	81.00	2.76	34.00
27	Career Choice	36	0.38	84.00	0.83	39.00

### Knowledge structure of the time period from 2004–2008

**Fig. 3:**
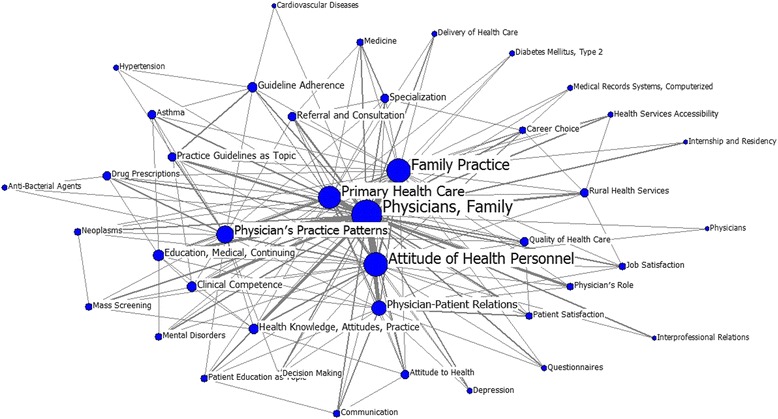
Map for keywords in GP research, 2004–2008. The size of nodes indicates the keywords centrality, and the thickness of the lines indicates the co-occurrence frequency of keywords pairs

A total of 41 high-frequency keywords were identified from research on GPs published from 2004–2008. “physicians, family” still tops the list with a frequency of 2424, followed by “attitude of health personnel,” “family practice,” “primary health care,” and “physician’s patient patterns.” Compared with other keywords illustrated in Table 5, these keywords have higher degree and betweenness values and lower closeness value. Accordingly, these five keywords above are in the central position of the social network in GPs research, and many paths that diversified from them connect to other keywords. Figure 3 also demonstrates that most research on GPs focus primarily on “physicians, family,” “family practice,” “attitude of health personnel,” and “primary health care.” However, “physician’s practice patterns” and “physician-patient relations” have received more attention based on the increasing frequency of new keywords linked to them.

**Table 5:** Individual centrality from 2004–2008

Rank	Major MeSH Terms	Frequency	Percentage (%)	Degree	Betweenness	Closeness
1	Physicians, Family	2424	17.33	3239.00	25.87	40.00
2	Attitude of Health Personnel	503	3.60	1017.00	25.87	40.00
3	Family Practice	495	3.54	840.00	24.38	41.00
4	Primary Health Care	455	3.25	732.00	23.30	41.00
5	Physician’s Practice Patterns	401	2.87	736.00	19.41	43.00
6	Physician–Patient Relations	277	1.98	528.00	19.50	44.00
7	Physician’s Role	148	1.06	237.00	8.66	52.00
8	Clinical Competence	144	1.03	282.00	13.47	47.00
9	Referral and Consultation	141	1.01	253.00	9.37	50.00
10	Health Knowledge, Attitudes, Practice	140	1.00	268.00	9.60	50.00
11	Education, Medical, Continuing	118	0.84	249.00	15.94	47.00
12	Rural Health Services	108	0.77	190.00	3.02	59.00
13	Practice Guidelines as Topic	99	0.71	221.00	3.16	59.00
14	Patient Satisfaction	87	0.62	184.00	5.83	55.00
15	Drug Prescriptions	84	0.60	154.00	4.45	58.00
16	Health Services Accessibility	80	0.57	113.00	2.98	61.00
17	Specialization	79	0.56	195.00	7.38	53.00
18	Communication	72	0.51	164.00	7.99	53.00
19	Delivery of Health Care	71	0.51	98.00	5.29	56.00
20	Mass Screening	69	0.49	143.00	5.06	55.00
21	Guideline Adherence	61	0.44	147.00	5.73	56.00
22	Quality of Health Care	57	0.41	109.00	3.24	59.00
23	Mental Disorders	57	0.41	113.00	3.09	58.00
24	Internship and Residency	57	0.41	88.00	1.77	62.00
25	Career Choice	56	0.40	127.00	1.94	63.00
26	Asthma	55	0.39	95.00	3.26	59.00
27	Neoplasms	55	0.39	104.00	1.01	62.00
28	Job Satisfaction	54	0.39	115.00	1.80	63.00
29	Attitude to Health	54	0.39	111.00	4.30	58.00
30	Patient Education as Topic	54	0.39	104.00	3.32	57.00
31	Medicine	53	0.38	150.00	3.67	59.00
32	Decision Making	52	0.37	105.00	11.01	51.00
33	Depression	51	0.36	90.00	2.22	61.00
34	Medical Records Systems, Computerized	49	0.35	70.00	1.04	65.00
35	Questionnaires	47	0.34	87.00	2.06	62.00
36	Hypertension	46	0.33	82.00	3.03	58.00
37	Anti-Bacterial Agents	45	0.32	81.00	0.87	65.00
38	Interprofessional Relations	42	0.30	80.00	4.27	59.00
39	Diabetes Mellitus, Type 2	41	0.29	63.00	3.00	61.00
40	Physicians	40	0.29	54.00	4.38	58.00
41	Cardiovascular Diseases	40	0.29	74.00	3.44	58.00

### Knowledge structure of the time period from 2009–2014

**Fig. 4:**
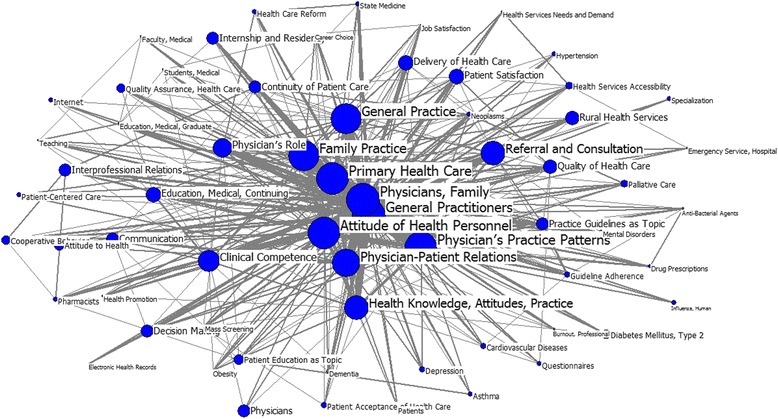
Map for keywords in GP research, 2009–2014. The size of nodes indicates the keywords centrality, and the thickness of the lines indicates the co-occurrence frequency of keywords pairs

A total of 64 high-frequency keywords were extracted from the papers published from 2009 to 2014. “General practitioner” replaces “family practice” in the rank of top four. Both “physicians, family” and “general practitioners” are placed exclusively at the central position in the social network of GP-related research; their degree values are similar, but much higher than those of the remaining keywords shown in Table 6. Moreover, “attitude of health personnel” and “primary health care” also play indispensable roles in the social network in accordance with their relatively high degrees and betweenness values. Because of their lowest closeness centrality values, “general practitioners,” and “physicians, family,” have the shortest commutation path with other keywords, which means that if any of these keywords is curtailed, the knowledge structure shown in Fig. 4 would show that GP-related research has undergone significant changes in recent years compared with former stages.

**Table 6:** Individual centrality from 2009–2014

Rank	Major MeSH Terms	Frequency	Percentage (%)	Degree	Betweenness	Closeness
1	General Practitioners	1727	8.99	2561.00	59.90	63.00
2	Physicians, Family	1688	8.79	2543.00	59.90	63.00
3	Attitude of Health Personnel	609	3.17	1326.00	57.54	64.00
4	Primary Health Care	608	3.16	1125.00	55.85	64.00
5	Physician′s Practice Patterns	527	2.74	1068.00	58.76	64.00
6	Family Practice	413	2.15	804.00	49.72	66.00
7	Physician-Patient Relations	310	1.61	701.00	42.21	69.00
8	General Practice	289	1.50	561.00	52.99	66.00
9	Health Knowledge, Attitudes, Practice	249	1.30	499.00	32.81	74.00
10	Clinical Competence	222	1.16	450.00	28.72	77.00
11	Physician′s Role	197	1.03	378.00	23.38	80.00
12	Referral and Consultation	178	0.93	362.00	33.29	74.00
13	Education, Medical, Continuing	137	0.71	285.00	15.35	86.00
14	Practice Guidelines as Topic	106	0.55	237.00	10.99	89.00
15	Delivery of Health Care	101	0.53	191.00	17.51	85.00
16	Neoplasms	99	0.52	190.00	3.61	99.00
17	Quality of Health Care	94	0.49	210.00	14.98	87.00
18	State Medicine	94	0.49	143.00	4.73	99.00
19	Patient Satisfaction	90	0.47	203.00	15.44	85.00
20	Communication	90	0.47	222.00	13.09	88.00
21	Rural Health Services	89	0.46	177.00	14.31	87.00
22	Health Services Accessibility	85	0.44	154.00	9.62	95.00
23	Continuity of Patient Care	81	0.42	181.00	11.77	89.00
24	Interprofessional Relations	80	0.42	193.00	9.94	89.00
25	Decision Making	79	0.41	161.00	10.51	89.00
26	Mass Screening	75	0.39	134.00	4.40	99.00
27	Health Care Reform	74	0.39	135.00	5.75	98.00
28	Guideline Adherence	72	0.37	172.00	7.53	96.00
29	Pharmacists	72	0.37	140.00	3.69	98.00
30	Drug Prescriptions	72	0.37	150.00	5.23	100.00
31	Mental Disorders	69	0.36	126.00	5.18	99.00
32	Depression	66	0.34	125.00	5.07	97.00
33	Internship and Residency	65	0.34	161.00	15.31	88.00
34	Education, Medical, Graduate	65	0.34	142.00	3.29	102.00
35	Diabetes Mellitus, Type 2	63	0.33	116.00	7.15	93.00
36	Patient Education as Topic	60	0.31	121.00	7.81	92.00
37	Palliative Care	58	0.30	126.00	4.89	95.00
38	Quality Assurance, Health Care	57	0.30	134.00	9.93	93.00
39	Cooperative Behavior	57	0.30	129.00	8.02	93.00
40	Cardiovascular Diseases	57	0.30	116.00	6.09	96.00
41	Career Choice	55	0.29	121.00	2.13	105.00
42	Asthma	54	0.28	103.00	3.48	98.00
43	Health Promotion	53	0.28	101.00	4.38	101.00
44	Patient Acceptance of Health Care	53	0.28	87.00	6.48	97.00
45	Hypertension	52	0.27	88.00	4.52	99.00
46	Teaching	50	0.26	117.00	2.943	102
47	Anti-Bacterial Agents	50	0.26	98.00	1.736	107
48	Health Services Needs and Demand	50	0.26	98.00	2.462	101
49	Questionnaires	50	0.26	95.00	3.93	98
50	Dementia	49	0.26	108.00	3.253	100
51	Burnout, Professional	48	0.25	87.00	1.536	108
52	Job Satisfaction	47	0.24	101.00	3.519	103
53	Attitude to Health	47	0.24	114.00	10.509	92
54	Obesity	47	0.24	105.00	2.257	103
55	Patient-Centered Care	45	0.23	96.00	5.864	98
56	Specialization	45	0.23	88.00	5.777	99
57	Influenza, Human	45	0.23	62.00	0.687	111
58	Emergency Service, Hospital	45	0.23	76.00	3.227	102
59	Physicians	44	0.23	88.00	14.455	88
60	Faculty, Medical	43	0.22	95.00	3.587	101
61	Internet	43	0.22	82.00	5.688	98
62	Students, Medical	43	0.22	110.00	4.547	101
63	Patients	42	0.22	98.00	3.681	101
64	Electronic Health Records	42	0.22	66.00	1.117	105

### Thematic evolution and trends analysis

**Fig. 5:**
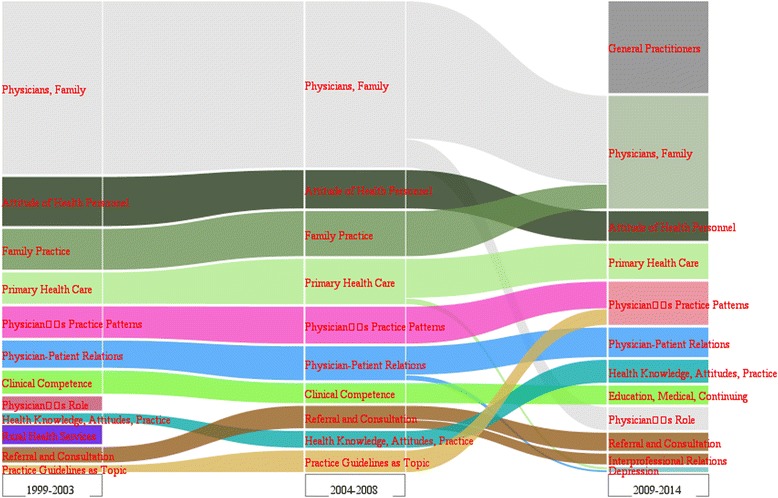
The evolution alluvial diagram of GP research

Figure 5 shows an overall picture of the topic evolution of research on GPs from 1999–2014, in which different bars of color represent different communities of GPs (themes or topics).

“Physicians, family” always topped the list of the diagram in all time periods (1999–2003, 2004–2008, and 2009–2014). The keywords “attitude of health personnel,” “family practice,” “general practitioner,” and “primary health care” also topped the diagram, indicating that they are the major topics of research on GPs. The keyword “attitude of health personnel” ranked fourth in 2009–2014 and involved a branch from “health knowledge, attitude, practice” in 2004–2008, which is also composed of partially “physician–patient patterns,” “physician’s role,” and “health knowledge, attitude, practice” communities in 1999–2003. Another branch of studies featuring “health knowledge, attitudes, practice” in 2004–2008 evolved a new community, that is, “mass screening” in 2009–2014. “General practitioner,” an emerging community back in 2009–2014, replaced the “attitude of health personnel” and became the second hot topic in the last period. This particular keyword relates to the evolution of keywords and changes depending on different concerns of considerable research on GPs. “Family practice” had a high spot in 1999–2003 and in 2004–2008. “Rural health service,” which emerged in the first period, merged into “family practice” community in 2004–2008, but finally disappeared and became a “death topic” in 2009–2014. A small branch from “primary health care” formed a new community, that is, “depression” in 2009–2014. Finally, the “physician–patient relations” community is divided into “physician–patient relations” and “patient satisfaction” in 2009–2014. The “referral and consultation” community in 1999–2003 and 2004–2008 introduced a new community, “inter-professional relations,” in 2009–2014. “Practice guidelines” as a topic community in 1999 to 2003 and 2004 to 2008 finally merged into the “physician’s practice patterns” community in 2009–2014.

## Discussions

### Publication trends of research on GPs

The publication trend of research on GPs is revealed to maintain a fluctuating increase with a slight decline occurring in 2004 before reaching its peak in 2008. The rapid increase of papers in 2004–2008 and the peak in 2008 might be related to the existence of health care reforms in most countries, which focused considerably on general practice and primary health care [47]. Although a decline trend of papers publication on GPs after 2008 was observed, in general, the total quantity of papers on GPs were still massive. Hence, higher numbers of better papers are expected to be produced in the impending maturity period. Moreover, compared with the fields of cardiovascular diseases, digestive system diseases, neoplasms and respiratory tract diseases, the gaps of absolute number and growth rate are increasing. General practice is a key discipline of primary care, and in many countries, GPs are physicians directly accessible to the public. The increase of research output pertaining to general practice can also promote the development of primary care. Although related research in this discipline shows a fluctuating increase, classical clinical disciplines, such as cardiovascular diseases, digestive system diseases, neoplasms and respiratory tract diseases have consistently been the focus of research. If the field of general practice intends to catch up, it will take many years of work and accumulated experience before this can happen [25].

### The knowledge structures of research on GPs

During the first stage, the knowledge structure could not be shaped if “physician, family” is removed (Fig. 2). Therefore, “physician, family” maintains its central position in the social network. The keywords “attitude of health personnel,” “family practice,” “physician’s practice patterns,” and “primary health care” also play vital roles in the social network of GP-related research. Consequently, these keywords have an indispensable place when information is transferred from one keyword to another, and can control information exchange among other keywords. “Physicians, family” is evidently the dominant keyword, but the other keywords related to it, such as “family practice,” “attitude of health personnel,” and “primary health care” are also at the center of the knowledge structure. The knowledge structure exists based on these main keywords. In general, research themes over this period aim mainly to sort and clarify the role/career orientation of GPs in primary care/family practice. The results demonstrate that research themes in this period focus mainly on the categories enumerated below. First, the basic role of GPs is within the scope of family practice/primary health care, such as conducting referrals, consultations, and mass screenings. The equity in the provision in rural health services is also considered a basic role of GPs. Second, the relationship between general practitioners/family physician and patients is a topic widely studied based on the keywords “physician-patient relations,” “communication,” and “patient education”. Third, with the development and changes in primary health and primary care teams, GPs now have opportunities to extend the range of their own skills and interests in clinical practice. Therefore, the development of GPs in terms of their clinical skills and career has received remarkable attention. Keywords “specialization,” “clinical competence,” and “education, medical, continuing” support such observation.

During the second stage, “physicians, family” still plays the central role, and “attitude of health personnel,” “family practice,” “primary health care,” and “physician’s patient patterns” are also in the top 5. This observation is similar with the previous finding in the first period; however, the knowledge structure shown in Fig. 3 is obviously more complex and scattered than the former period. In this period, the basic role of GPs in primary health care/family practice remains the main focus of research on GPs. However, such works have begun to involve the quality and accessibility of primary health services with new emerging keywords (i.e., “health services accessibility,” “delivery of health care,” and “quality of health care”) shown in Table 5. The role of GPs in managing diseases, especially in managing chronic diseases (e.g., asthma, hypertension, diabetes mellitus type 2, cardiovascular diseases, mental disorders, and depression) is also fast becoming an emergent research topic in this field. This may be because during this period, most Western countries (e.g., Australia, the US, and New Zealand) spent significant amount of time refocusing their health care systems to address the increasing burden of chronic diseases [48, 49]. The CP–patient relation and clinical development of GPs (i.e., continuing education and clinical competence improvement) are also considered main research themes from 2004–2008. Moreover, literature on GPs has begun to emphasize the spiritual/psychological experience of both GPs and patients according to the keywords “patient” and “job satisfaction.” Based on the above analysis, substantial amount of research on GPs published in this period are more in-depth and diversified than works published in the last stage. Moreover, during these years, GPs have begun to provide patients with higher-quality, more equitable, and comprehensive health services. A new keyword, “medical records systems, computerized” also emerged during this period, indicating that the use of computers and computerized medical records is becoming more popular in primary health care services. Hence, the topics related to “medical records systems, computerized” and GPs are also widely investigated.

Unlike in the last two periods, during the third stage, “general practitioner” replaces “family practice” in the rank of top four, and both “physicians, family” and “general practitioners” are placed at the central position. Both Table 6 and Fig. 4 indicate that research themes can be clustered into several respects. The first one pertains to the new role changes of GPs in primary/family/general practices that correspond with health care reforms throughout the world. The new keyword “general practice,” which refers mainly to community-orientation, has received wide attention because of the increasing consideration paid to primary health care. Hence, GPs have gradually provided person-centered, continuing, comprehensive, and coordinated whole person health care services to individuals and families in their respective communities [47]. The second aspect refers to the comprehensive functions of GPs. Other than focusing on managing chronic diseases, in this period, the GPs are more concerned with health promotion and disease follow-up care to meet the challenges that confront the reformed health care system, including the existence of an ageing population accompanied by an increased prevalence of long-term health conditions. The third aspect relates to care coordination with other types of health and social care providers. The whole world is confronted with problems regarding chronic and complex disease management. Thus, closer inter-professional cooperation is urgently needed [50]. In this context, GPs and other relevant persons involved must work together as part of a healthcare team that provides the best health care practice [51, 52].

### The thematic evolution of research on GPs

Overview, the majority of the themes maintain stable positions or only undergo slight rank changes during the former two stages. The thematic evolution results show that many research topics do not emerge out of the void and are more or less associated with the presence of other topics that emerged in the past. Many themes are based on previous scientific research and are produced gradually, although different forms of thematic evolution have taken place in recent years as shown in Fig. 5. For example, the “general practitioners” community in 2009–2014 emerged with the development of primary care and the evolution of general practice. Specifically, with the enhancement and improvement of primary health care, general practice has become a place (both real and virtual) of comprehensive health service, in which individual patients are not only provided with episodic care and ongoing clinical management, but also granted access to preventive care, health education, and other services. The focus of primary health care [53] has been widely analyzed in the literature, which corresponds to an increase in works that study the roles of GPs in general practice. “Inter-professional relations,” a new community that emerged in the 2009–2014 period, developed from the “referral and consultation” community in the last two periods. Such an occurrence may be because of the improvement of primary health care, inter-professional relations with broader connotation and referral and consultation, which gradually formed a new community in 2009–2014. Pieces of evidence indicate that high-quality community-based palliative care is achieved with effective multidisciplinary teamwork, good inter-professional relationships (i.e., good communication between GPs and district nurses), and early referral of patients to district nurses [54]. In the last two decades of the 20^th^ century, the prevalence of depression in the general population has constituted a major health burden among developed countries, and an increase in recognizing the importance of ensuring its identification and treatment in primary health care has been observed [55, 56]. Therefore, with thematic evolution, “depression” finally became a new community from the “primary health care” expansion in 2009–2014. In summary, the considerable amount of research on GPs has great potential for further development.

### Hot topics found in research on GPs

#### Multiple roles and competency improvement of GPs

Except for the foundational role of GPs in primary health care and with the represented keywords (e.g., “referral and consultation,” “drug prescriptions,” “delivery of health care,” “mass screening,” and “patient education as topic” shown from 1999–2014), GPs have continuously played important roles in primary care management, improving quality and ensuring equity of primary health care services, which has become a major concern as a research topic. Anne et al. conducted research to assess the geographical equity in the availability and accessibility of GP services for women in Australia, and their analysis results indicated that women living in rural areas gave lower ratings for availability, accessibility, and affordability of GP services than women in urban areas [57]. The new trends of knowledge structure analysis reveal that GPs have gradually provided person-centered, community-orientation health care services with comprehensive approaches. The management of chronic diseases has also become one of the most important tasks of GPs. The importance of GPs in managing patients with chronic diseases, especially asthma, hypertension, Type 2 diabetes, and mental health issues, include the initial diagnosis, initiating treatment, risk factor interventions to overall continuity of care. Many studies have explored the disease management of patients with specific populations, such as women [58], older adults [59–62], and children [63]. The role of GPs in promoting health and preventing disease, whether effective or not, must be investigated further.

As for the competency of GPs, continuous improvements involving education level, specific problem-solving competence, and communication skill with patients have become the focus of studies on GPs in response to the increasing needs of quality improvement in primary care in many countries. In England, some GPs have taken leading roles in their practices for specific clinical areas [64]. GPs are also gaining more specific problem-solving skills. Joanna et al. defined GPs as physicians who supplement their generalist role by delivering high quality and improved accessibility to services. Dermatology and respiratory diseases are areas that GPs with special interest have chosen to develop in recent years [65, 66]. With healthcare system reforms as well as the problem-solving and skills development of GPs, more specific themes on the role and competencies of GPs may become available to meet the challenges that confront disease management. Such challenges include new concepts of patient empowerment and continuous quality improvement, which has been revised in the 2011 edition of European Definition of General Practice/Family Medicine compared with the 2002 and 2005 edition [67].

## Conclusions

Scientometrics, co-word analysis, and social network analysis were combined and used to reveal the knowledge structures and thematic evolution of research on GPs published from 1999–2014. The number of studies on GPs has rapidly increased but the growth rate has decreased to some extent. The gaps between GPs and others (e.g., cardiovascular diseases, digestive system diseases, neoplasms, and respiratory tract diseases) have also been growing in recent years.

The research on GPs varies and develops with the changes in health care reforms, health policies, and functions of GPs in many countries, especially in recent years. The multiple roles and competency improvement of GPs, as well as the relations between GPs and patients/others involved (e.g., health care providers) have reflected the core competencies of GPs, especially in primary care management, person-centered care, specific problem solving skills and community-orientation, in accordance with the European Definition of General Practice/Family Medicine [67]. More substantial research, especially on comprehensive approaches, olistic modeling, patient empowerment, and continuous quality improvement should be accomplished. This study also anticipates that, owing to the growth of the elderly population, elderly persons shall be the main topics of the major specific groups in GP-related research.

### Limitations

First, literature was extracted only from the PubMed database, which may not contain all literature related to GP research, especially non-English articles and some grey literature (e.g., reports or internal materials). Second, only high-frequency words were analyzed; hence, the results could only show the hot topics on GP-related research. Some new emerging topics with low attention may not have been shown in the map. Therefore, analyses combining multiple databases and new emerging topics should be conducted in future studies.

## Abbreviations

GPs: General practitioners
BICOMB: Bibliographic item co-occurrence matrix builder
RGR: relative growth rate
Dt: double time
